# 14,15-Didehydro­hellebrigenin

**DOI:** 10.1107/S1600536812018570

**Published:** 2012-05-05

**Authors:** Tong Yu, Hai-Yan Tian, Xiao-Feng Yuan, Shu-Zhi Hu, Ren-Wang Jiang

**Affiliations:** a Guangdong Province Key Laboratory of Pharmacodynamic Constituents of Traditional Chinese Medicine and New Drugs Research, Institute of Traditional Chinese Medicine and Natural Products, Jinan University, Guangzhou 510632, People’s Republic of China

## Abstract

The title compound, C_24_H_30_O_5_, is the didehydro product of the steroid hellebrigenin (systematic name: 3β,5,14-trihy­droxy-19-oxo-5β-bufa-20,22-dienolide). It consists of three cyclo­hexane rings (*A*, *B* and *C*), a five-membered ring (*D*) and a six-membered lactone ring (*E*). The stereochemistry of the ring junctions are *A*/*B cis*, *B*/*C trans* and *C*/*D cis*. Cyclo­hexane rings *A*, *B* and *C* have normal chair conformations. The five-membered ring *D* with the C=C bond adopts an envelope conformation. Lactone ring *E* is essentially planar with a mean derivation of 0.006 (4) Å and is β-oriented at the C atom of ring *D* to which it is attached. There is an O—H⋯O hydrogen bond in the mol­ecule involving the hy­droxy groups. In the crystal, O—H⋯O hydrogen bonds link the mol­ecules into chains propagating along [010]. The chains are linked by C—H⋯O contacts into a three-dimensional network.

## Related literature

For previous isolations of hellebrigenin, see: Urscheler *et al.* (1955 ▶); Yang *et al.* (2010 ▶); Zhao *et al.* (2010 ▶). For its inhibitory activity against adenosinetriphosphatase of the 3-acetate, 3,5-diacetate, 3-iodo­acetate and 3-bromo­acetate of hellebrigenin, see: Ruoho *et al.* (1968 ▶). For the treatment of hellebrigenin with sodium hydroxide, see: Kupchan *et al.* (1969 ▶). For the stereochemistry of bufalin and secohellebrigeninamide, see: Rohrer *et al.* (1982 ▶); Yuan *et al.* (2012 ▶).
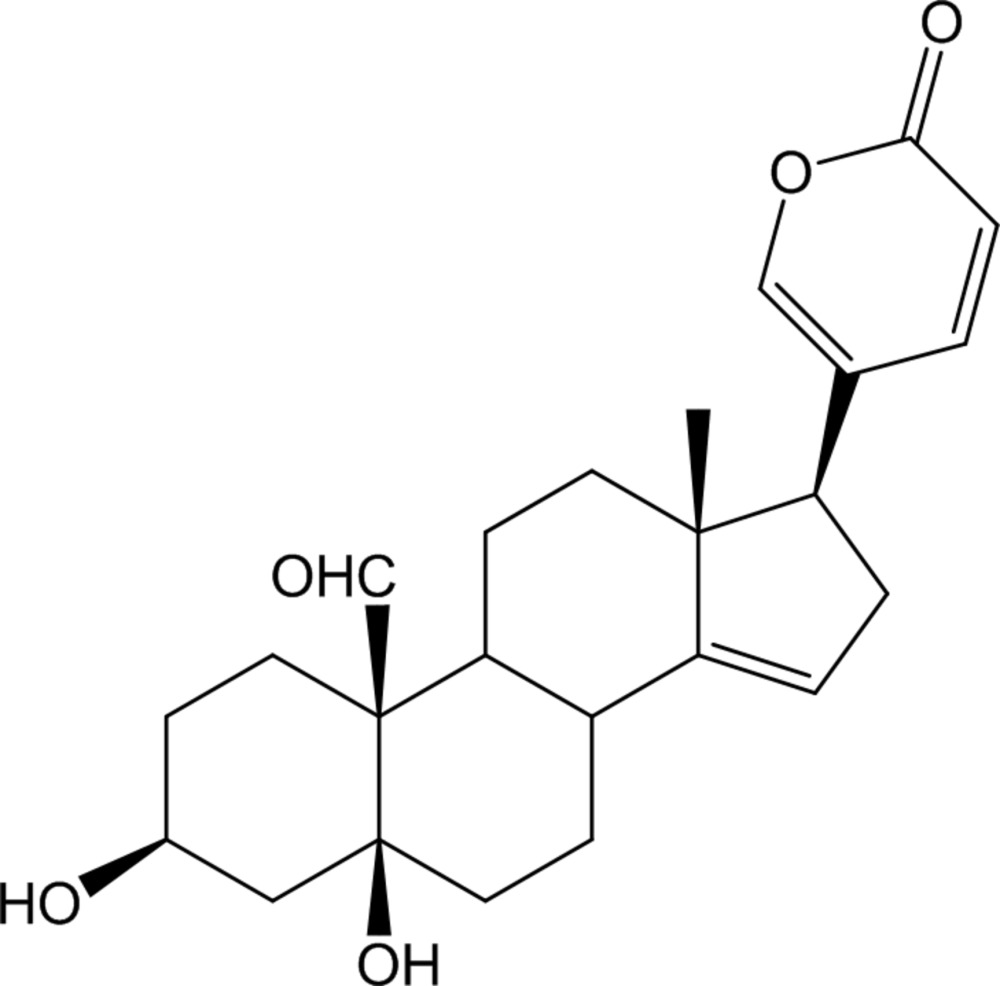


## Experimental

### 

#### Crystal data

C_24_H_30_O_5_
*M* *_r_* = 398.48
Monoclinic, 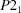

*a* = 10.7628 (4) Å
*b* = 6.6016 (2) Å
*c* = 14.6376 (5) Å
β = 94.224 (3)°
*V* = 1037.20 (6) Å^3^
*Z* = 2
Cu *K*α radiation
μ = 0.71 mm^−1^
*T* = 291 K
0.40 × 0.26 × 0.23 mm

#### Data collection

Oxford Gemini S Ultra Sapphire CCD diffractometer
Absorption correction: multi-scan (*CrysAlis PRO*; Agilent, 2011 ▶) *T*_min_ = 0.819, *T*_max_ = 1.000
3100 measured reflections
2238 independent reflections
2042 reflections with *I* > 2σ(*I*)
*R*_int_ = 0.023

#### Refinement

*R*[*F* ^2^ > 2σ(*F* ^2^)] = 0.042
*wR*(*F* ^2^) = 0.115
*S* = 1.04
2238 reflections
266 parameters
1 restraint
H-atom parameters constrained
Δρ_max_ = 0.24 e Å^−3^
Δρ_min_ = −0.20 e Å^−3^

### 

Data collection: *CrysAlis PRO* (Agilent, 2011 ▶); cell refinement: *CrysAlis PRO*; data reduction: *XPREP* (Sheldrick, 2008 ▶); program(s) used to solve structure: *SHELXS97* (Sheldrick, 2008 ▶); program(s) used to refine structure: *SHELXL97* (Sheldrick, 2008 ▶); molecular graphics: *XP* in *SHELXTL* (Sheldrick, 2008 ▶); software used to prepare material for publication: *SHELXTL*.

## Supplementary Material

Crystal structure: contains datablock(s) I, global. DOI: 10.1107/S1600536812018570/su2412sup1.cif

Structure factors: contains datablock(s) I. DOI: 10.1107/S1600536812018570/su2412Isup2.hkl

Additional supplementary materials:  crystallographic information; 3D view; checkCIF report

## Figures and Tables

**Table 1:** Hydrogen-bond geometry (Å, °)

*D*—H⋯*A*	*D*—H	H⋯*A*	*D*⋯*A*	*D*—H⋯*A*
O2—H2⋯O1	0.82	2.05	2.773 (3)	147
O1—H1⋯O2^i^	0.82	2.01	2.786 (3)	158
C9—H9⋯O3^ii^	0.98	2.58	3.545 (4)	169
C15—H15⋯O4^iii^	0.93	2.58	3.447 (4)	155
C22—H22⋯O4^iv^	0.93	2.60	3.233 (6)	126

Symmetry codes: (i) 
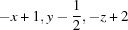
; (ii) 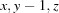; (iii) 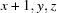; (iv) 
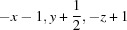
.

**Fig. 1.**
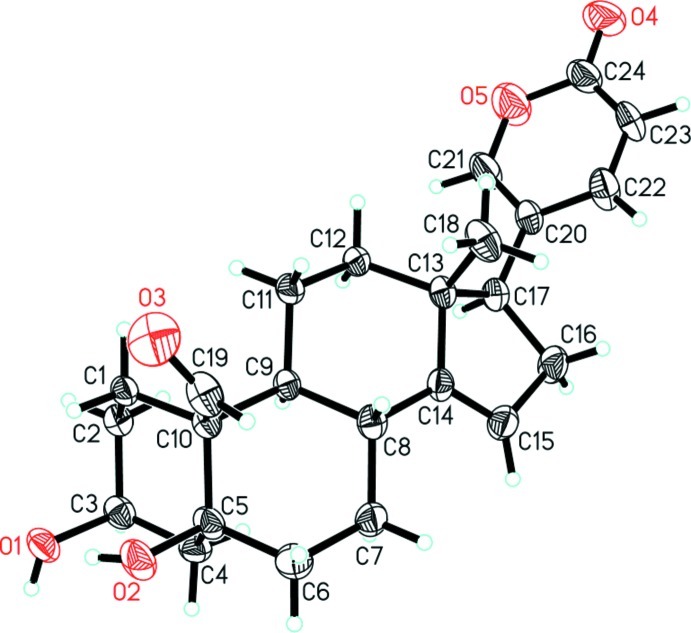
The molecular structure of the title molecule with the atom numbering. The displacement ellipsoids are drawn at the 30% probability level.

**Fig. 2.**
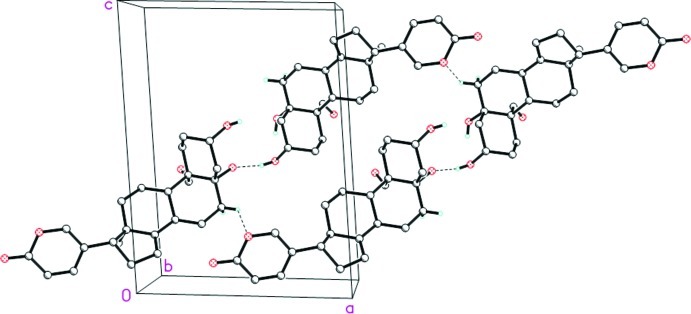
A view along the b axis of the crystal packing of the title compound, showing the intermolecular O—H···O hydrogen bonds and C—H···O short contacts (dashed lines; only selected H-atoms highlighting the hydrogen bondings and short contacts are shown; see Table 1 for details).

## supplementary crystallographic information

### Comment

Hellebrigenin is a cardiac steroid that exists in the skin of toads (Urscheler, *et al.*, 1955; Zhao, *et al.*, 2010)) and the rhizomes of Helleborus thibetanus (Yang, *et al.*, 2010). It is an inhibitor of adenosinetriphosphatase. The 3-acetate, 3,5-diacetate, 3-iodoacetate and 3-bromoacetate of hellebrigenin were shown to be potent irreversible inhibitors of the enzyme (Ruoho, *et al.*, 1968). The lactone ring at atom C17 is not stable at base conditions. Treatment of hellebrigenin with sodium hydroxide in methanol afforded methyl isohellebrigeninate (Kupchan, *et al.*, 1969). While treatment of hellebrigenin with *N*,*N*-dimethylformamide (DMF) resulted in a derivative named as secohellebrigeninamide (Yuan *et al.*, 2012). Recently, hellebrigenin was treated with hydrochloric acid and the new derivative 14,15-didehydro hellebrigenin was obtained, and we report herein on its crystal structure.

The title molecule (Fig. 1) is composed of three cyclohexane rings (A, B and C), an unsaturated five-membered ring (D) and a six-membered lactone ring (E). The stereochemistry of the ring junctions are A/B *cis*, B/C *trans* and C/D *cis*. The cyclohexane rings A, B and C have normal chair conformations. The unsaturated five-membered ring D adopts an envelope conformation. The lactone ring E is planar with a mean derivation of 0.006 (4) Å and is β-oriented at C17 in ring D. The mean planes of the lactone ring E and ring D (atoms C13/C14/C15/C16) make a dihedral angle of 59.6 (1)°. There is an O-H···O hydrogen bond in the molecule involving the hydroxyl groups (Table 1).

In the crystal, O—H···O hydrogen bonds between the hydroxyl group at C3 and the hydroxyl group at C5 link adjacent molecules into dimers (Fig 2 and Table 1). Adjacent dimers are linked by short C–H···O contacts, between the methylene group at C6 and the lactone group, to form a three-dimensional network (Table 1 and Fig. 2).

The absolute configuration determined for bufalin (Rohrer *et al.*, 1982) and secohellebrigeninamide, (Yuan *et al.*, 2012), two similar steroids, were invoked, giving the assignments of the chiral centres in the molecule as shown in Fig. 1.

### Experimental

Hellebrigenin (104.32 mg) was dissolved in a mixture of dichloromethane (2 ml) and methanol (3 ml). Hydrochloric acid (36%, 10 ml) was added. The solution was stirred for three hours at room temperature. Then the mixture was then poured into cold water and extracted with ethyl acetate. The organic phase was washed twice with water and concentrated under reduced pressure. The residue was subjected to preparative HPLC to afford the title compound (41 mg). The compound was recrystallized in methanol at room temperature to afford colourless prism-like crystals.

### Refinement

The C-bound H-atoms were included in calculated positions and treated as riding atoms: O-H = 0.82 Å, C-H = 0.98, 0.96, 0.97 and 0.93 Å for CH, CH_3_, CH_2_ and CH(aryl) H-atoms, respectively, with U_iso_(H) = k × U_eq_(O,C), where k = 1.5 for OH and CH_3_ H-atoms and = 1.2 for other H-atoms. The absolute configuration of the title compound could not be determined crystallographically; the Flack parameter refined to -0.1 (3). It was assigned with reference to the known configuration of the closely related compounds bufalin (Rohrer *et al.*, 1982) and secohellebrigeninamide (Yuan *et al.*, 2012).

### Crystal data

**Table:**

C_24_H_30_O_5_	*F*(000) = 428
*M**_r_* = 398.48	*D*_x_ = 1.276 Mg m^−^^3^
Monoclinic, *P*2_1_	Cu *K*α radiation, λ = 1.54184 Å
*a* = 10.7628 (4) Å	Cell parameters from 1435 reflections
*b* = 6.6016 (2) Å	θ = 3.0–62.5°
*c* = 14.6376 (5) Å	µ = 0.71 mm^−^^1^
β = 94.224 (3)°	*T* = 291 K
*V* = 1037.20 (6) Å^3^	Prism, colourless
*Z* = 2	0.40 × 0.26 × 0.23 mm

### Data collection

**Table:**

Oxford Gemini S Ultra Sapphire CCD diffractometer	2238 independent reflections
Radiation source: fine-focus sealed tube	2042 reflections with *I* > 2σ(*I*)
Graphite monochromator	*R*_int_ = 0.023
ω scan	θ_max_ = 62.6°, θ_min_ = 3.0°
Absorption correction: multi-scan (*CrysAlis PRO*; Agilent, 2011)	*h* = −6→12
*T*_min_ = 0.819, *T*_max_ = 1.000	*k* = −7→4
3100 measured reflections	*l* = −16→16

### Refinement

**Table:**

Refinement on *F*^2^	Secondary atom site location: difference Fourier map
Least-squares matrix: full	Hydrogen site location: inferred from neighbouring sites
*R*[*F*^2^ > 2σ(*F*^2^)] = 0.042	H-atom parameters constrained
*wR*(*F*^2^) = 0.115	*w* = 1/[σ^2^(*F*_o_^2^) + (0.0726*P*)^2^ + 0.1057*P*] where *P* = (*F*_o_^2^ + 2*F*_c_^2^)/3
*S* = 1.04	(Δ/σ)_max_ < 0.001
2238 reflections	Δρ_max_ = 0.24 e Å^−^^3^
266 parameters	Δρ_min_ = −0.20 e Å^−^^3^
1 restraint	Extinction correction: *SHELXL97* (Sheldrick, 2008), Fc^*^=kFc[1+0.001xFc^2^λ^3^/sin(2θ)]^-1/4^
Primary atom site location: structure-invariant direct methods	Extinction coefficient: 0.0045 (12)

### Special details

**Table:**

Geometry. Bond distances, angles etc. have been calculated using the rounded fractional coordinates. All su's are estimated from the variances of the (full) variance-covariance matrix. The cell esds are taken into account in the estimation of distances, angles and torsion angles
Refinement. Refinement of *F*^2^ against ALL reflections. The weighted *R*-factor *wR* and goodness of fit *S* are based on *F*^2^, conventional *R*-factors *R* are based on *F*, with *F* set to zero for negative *F*^2^. The threshold expression of *F*^2^ > σ(*F*^2^) is used only for calculating *R*-factors(gt) *etc*. and is not relevant to the choice of reflections for refinement. *R*-factors based on *F*^2^ are statistically about twice as large as those based on *F*, and *R*- factors based on ALL data will be even larger.

### Fractional atomic coordinates and isotropic or equivalent isotropic displacement parameters (Å^2^)

**Table:**

	*x*	*y*	*z*	*U*_iso_*/*U*_eq_	
O1	0.40415 (18)	0.7669 (4)	1.06950 (14)	0.0564 (7)	
O2	0.3968 (2)	0.9967 (4)	0.91061 (15)	0.0605 (8)	
O3	0.1076 (3)	1.2425 (4)	0.8935 (2)	0.0882 (11)	
O4	−0.6574 (2)	0.2486 (7)	0.5959 (2)	0.0998 (13)	
O5	−0.4844 (3)	0.2651 (7)	0.69025 (19)	0.1009 (13)	
C1	0.1594 (2)	0.8911 (5)	0.98577 (16)	0.0395 (8)	
C2	0.1910 (3)	0.6866 (5)	1.02785 (18)	0.0438 (9)	
C3	0.3250 (3)	0.6280 (5)	1.01530 (18)	0.0478 (9)	
C4	0.3517 (3)	0.6385 (5)	0.91490 (19)	0.0475 (9)	
C5	0.3174 (2)	0.8405 (5)	0.86880 (19)	0.0451 (9)	
C6	0.3428 (3)	0.8346 (7)	0.7677 (2)	0.0609 (13)	
C7	0.2526 (3)	0.6979 (6)	0.71152 (19)	0.0552 (10)	
C8	0.1166 (2)	0.7489 (5)	0.72275 (17)	0.0435 (9)	
C9	0.0893 (2)	0.7573 (5)	0.82503 (16)	0.0358 (8)	
C10	0.1798 (2)	0.9001 (4)	0.88277 (16)	0.0376 (8)	
C11	−0.0483 (2)	0.8023 (5)	0.83501 (19)	0.0438 (9)	
C12	−0.1299 (2)	0.6349 (5)	0.79095 (17)	0.0427 (9)	
C13	−0.1110 (2)	0.6065 (5)	0.68901 (16)	0.0390 (8)	
C14	0.0279 (2)	0.6028 (5)	0.67349 (16)	0.0414 (8)	
C15	0.0524 (3)	0.4602 (6)	0.61287 (19)	0.0544 (10)	
C16	−0.0631 (3)	0.3465 (6)	0.5790 (2)	0.0597 (10)	
C17	−0.1499 (3)	0.3905 (5)	0.65465 (18)	0.0439 (9)	
C18	−0.1773 (3)	0.7733 (6)	0.6318 (2)	0.0599 (11)	
C19	0.1572 (3)	1.1166 (5)	0.8511 (2)	0.0560 (11)	
C20	−0.2876 (3)	0.3558 (5)	0.63458 (18)	0.0459 (9)	
C21	−0.3594 (3)	0.3021 (8)	0.7029 (2)	0.0707 (13)	
C22	−0.3499 (3)	0.3797 (7)	0.5489 (2)	0.0683 (13)	
C23	−0.4722 (3)	0.3462 (7)	0.5348 (2)	0.0660 (13)	
C24	−0.5468 (3)	0.2853 (7)	0.6046 (3)	0.0716 (13)	
H1	0.47040	0.71120	1.08520	0.0850*	
H1A	0.21060	0.99380	1.01750	0.0470*	
H1B	0.07300	0.92230	0.99440	0.0470*	
H2	0.40780	0.97540	0.96580	0.0910*	
H2A	0.13570	0.58490	0.99960	0.0530*	
H2B	0.17820	0.69050	1.09270	0.0530*	
H3	0.34000	0.48970	1.03780	0.0570*	
H4A	0.30580	0.53130	0.88210	0.0570*	
H4B	0.43970	0.61350	0.90990	0.0570*	
H6A	0.33670	0.97090	0.74300	0.0730*	
H6B	0.42710	0.78710	0.76200	0.0730*	
H7A	0.26790	0.55840	0.72980	0.0660*	
H7B	0.26840	0.70970	0.64730	0.0660*	
H8	0.10020	0.88370	0.69650	0.0520*	
H9	0.10440	0.62050	0.84930	0.0430*	
H11A	−0.06290	0.81300	0.89940	0.0530*	
H11B	−0.07020	0.93080	0.80600	0.0530*	
H12A	−0.21660	0.66700	0.79790	0.0510*	
H12B	−0.11100	0.50860	0.82290	0.0510*	
H15	0.13140	0.43410	0.59380	0.0650*	
H16A	−0.04700	0.20260	0.57370	0.0720*	
H16B	−0.09700	0.39790	0.52020	0.0720*	
H17	−0.12410	0.29820	0.70490	0.0530*	
H18A	−0.26480	0.76990	0.64050	0.0900*	
H18B	−0.16490	0.75220	0.56830	0.0900*	
H18C	−0.14390	0.90280	0.65080	0.0900*	
H19	0.18410	1.15340	0.79450	0.0670*	
H21	−0.32140	0.28980	0.76170	0.0850*	
H22	−0.30540	0.42010	0.49990	0.0820*	
H23	−0.50960	0.36410	0.47610	0.0790*	

### Atomic displacement parameters (Å^2^)

**Table:**

	*U*^11^	*U*^22^	*U*^33^	*U*^12^	*U*^13^	*U*^23^
O1	0.0446 (11)	0.0602 (15)	0.0613 (12)	0.0074 (11)	−0.0164 (9)	−0.0076 (12)
O2	0.0543 (12)	0.0632 (16)	0.0627 (12)	−0.0261 (12)	−0.0045 (10)	−0.0051 (12)
O3	0.116 (2)	0.0359 (15)	0.112 (2)	0.0128 (16)	0.0031 (17)	0.0009 (16)
O4	0.0522 (14)	0.124 (3)	0.120 (2)	−0.0018 (18)	−0.0148 (14)	−0.038 (2)
O5	0.0697 (16)	0.150 (3)	0.0816 (17)	−0.025 (2)	−0.0037 (13)	0.007 (2)
C1	0.0372 (13)	0.0389 (16)	0.0414 (13)	−0.0009 (12)	−0.0037 (10)	−0.0075 (13)
C2	0.0453 (15)	0.0443 (18)	0.0412 (13)	−0.0040 (13)	−0.0007 (11)	0.0019 (13)
C3	0.0476 (15)	0.0405 (18)	0.0535 (15)	0.0029 (14)	−0.0081 (12)	−0.0001 (14)
C4	0.0372 (14)	0.0475 (19)	0.0573 (15)	0.0057 (13)	0.0012 (11)	−0.0085 (15)
C5	0.0384 (14)	0.0475 (19)	0.0486 (14)	−0.0148 (14)	−0.0012 (11)	−0.0030 (14)
C6	0.0495 (16)	0.080 (3)	0.0543 (16)	−0.0202 (18)	0.0106 (13)	0.0022 (18)
C7	0.0486 (16)	0.075 (2)	0.0433 (14)	−0.0173 (16)	0.0113 (11)	−0.0056 (16)
C8	0.0496 (15)	0.0422 (18)	0.0382 (13)	−0.0053 (14)	0.0008 (11)	0.0031 (13)
C9	0.0392 (13)	0.0307 (15)	0.0370 (12)	−0.0043 (12)	−0.0011 (9)	−0.0034 (12)
C10	0.0413 (13)	0.0304 (15)	0.0404 (13)	−0.0012 (12)	−0.0026 (10)	−0.0007 (12)
C11	0.0411 (14)	0.0432 (18)	0.0458 (14)	0.0023 (13)	−0.0048 (11)	−0.0111 (13)
C12	0.0375 (13)	0.0494 (18)	0.0408 (13)	−0.0058 (13)	0.0007 (10)	−0.0094 (14)
C13	0.0458 (14)	0.0342 (16)	0.0361 (12)	−0.0023 (13)	−0.0039 (10)	−0.0006 (12)
C14	0.0467 (14)	0.0467 (18)	0.0306 (11)	−0.0092 (14)	0.0020 (10)	0.0004 (13)
C15	0.0555 (17)	0.061 (2)	0.0475 (15)	−0.0072 (17)	0.0087 (12)	−0.0118 (17)
C16	0.0653 (18)	0.062 (2)	0.0520 (15)	−0.0126 (18)	0.0053 (14)	−0.0218 (16)
C17	0.0509 (15)	0.0412 (17)	0.0388 (12)	−0.0044 (14)	−0.0010 (11)	−0.0025 (13)
C18	0.069 (2)	0.0444 (19)	0.0628 (18)	−0.0022 (17)	−0.0196 (14)	0.0090 (17)
C19	0.069 (2)	0.0352 (18)	0.0610 (17)	−0.0103 (17)	−0.0131 (15)	0.0044 (16)
C20	0.0526 (15)	0.0406 (17)	0.0431 (13)	−0.0074 (15)	−0.0064 (12)	−0.0031 (14)
C21	0.0501 (17)	0.106 (3)	0.0540 (17)	−0.021 (2)	−0.0095 (13)	0.010 (2)
C22	0.072 (2)	0.082 (3)	0.0487 (16)	−0.021 (2)	−0.0097 (15)	0.0023 (19)
C23	0.0618 (19)	0.079 (3)	0.0527 (16)	−0.0093 (19)	−0.0253 (15)	−0.0017 (18)
C24	0.0517 (18)	0.079 (3)	0.081 (2)	0.002 (2)	−0.0169 (16)	−0.020 (2)

### Geometric parameters (Å, º)

**Table:**

O1—C3	1.448 (4)	C20—C22	1.387 (4)
O2—C5	1.446 (4)	C22—C23	1.336 (5)
O3—C19	1.187 (4)	C23—C24	1.404 (5)
O4—C24	1.212 (4)	C1—H1A	0.9700
O5—C21	1.367 (5)	C1—H1B	0.9700
O5—C24	1.384 (5)	C2—H2A	0.9700
O1—H1	0.8200	C2—H2B	0.9700
O2—H2	0.8200	C3—H3	0.9800
C1—C10	1.541 (3)	C4—H4A	0.9700
C1—C2	1.512 (4)	C4—H4B	0.9700
C2—C3	1.517 (5)	C6—H6A	0.9700
C3—C4	1.520 (4)	C6—H6B	0.9700
C4—C5	1.528 (5)	C7—H7A	0.9700
C5—C10	1.560 (3)	C7—H7B	0.9700
C5—C6	1.525 (4)	C8—H8	0.9800
C6—C7	1.522 (5)	C9—H9	0.9800
C7—C8	1.523 (4)	C11—H11A	0.9700
C8—C9	1.548 (3)	C11—H11B	0.9700
C8—C14	1.503 (4)	C12—H12A	0.9700
C9—C10	1.559 (4)	C12—H12B	0.9700
C9—C11	1.528 (3)	C15—H15	0.9300
C10—C19	1.517 (4)	C16—H16A	0.9700
C11—C12	1.525 (4)	C16—H16B	0.9700
C12—C13	1.532 (3)	C17—H17	0.9800
C13—C14	1.529 (3)	C18—H18A	0.9600
C13—C17	1.559 (5)	C18—H18B	0.9600
C13—C18	1.528 (5)	C18—H18C	0.9600
C14—C15	1.333 (4)	C19—H19	0.9300
C15—C16	1.505 (5)	C21—H21	0.9300
C16—C17	1.528 (4)	C22—H22	0.9300
C17—C20	1.507 (5)	C23—H23	0.9300
C20—C21	1.355 (4)		
			
C21—O5—C24	120.8 (3)	C3—C2—H2A	109.00
C3—O1—H1	110.00	C3—C2—H2B	109.00
C5—O2—H2	109.00	H2A—C2—H2B	108.00
C2—C1—C10	112.9 (2)	O1—C3—H3	109.00
C1—C2—C3	111.4 (2)	C2—C3—H3	109.00
O1—C3—C2	107.4 (2)	C4—C3—H3	109.00
O1—C3—C4	110.8 (3)	C3—C4—H4A	109.00
C2—C3—C4	110.8 (2)	C3—C4—H4B	109.00
C3—C4—C5	114.2 (3)	C5—C4—H4A	109.00
O2—C5—C10	107.6 (2)	C5—C4—H4B	109.00
C4—C5—C6	110.6 (3)	H4A—C4—H4B	108.00
C4—C5—C10	111.3 (2)	C5—C6—H6A	109.00
C6—C5—C10	111.9 (2)	C5—C6—H6B	109.00
O2—C5—C6	106.6 (2)	C7—C6—H6A	109.00
O2—C5—C4	108.6 (2)	C7—C6—H6B	109.00
C5—C6—C7	112.8 (3)	H6A—C6—H6B	108.00
C6—C7—C8	113.0 (3)	C6—C7—H7A	109.00
C9—C8—C14	109.1 (2)	C6—C7—H7B	109.00
C7—C8—C9	111.4 (2)	C8—C7—H7A	109.00
C7—C8—C14	112.8 (3)	C8—C7—H7B	109.00
C8—C9—C10	113.0 (2)	H7A—C7—H7B	108.00
C8—C9—C11	110.72 (19)	C7—C8—H8	108.00
C10—C9—C11	113.7 (2)	C9—C8—H8	108.00
C1—C10—C5	108.88 (19)	C14—C8—H8	108.00
C5—C10—C9	109.7 (2)	C8—C9—H9	106.00
C5—C10—C19	109.3 (2)	C10—C9—H9	106.00
C9—C10—C19	108.9 (2)	C11—C9—H9	106.00
C1—C10—C9	112.3 (2)	C9—C11—H11A	110.00
C1—C10—C19	107.7 (2)	C9—C11—H11B	110.00
C9—C11—C12	110.5 (2)	C12—C11—H11A	110.00
C11—C12—C13	112.8 (2)	C12—C11—H11B	110.00
C12—C13—C17	112.0 (2)	H11A—C11—H11B	108.00
C12—C13—C14	110.37 (18)	C11—C12—H12A	109.00
C14—C13—C18	110.6 (2)	C11—C12—H12B	109.00
C17—C13—C18	112.3 (2)	C13—C12—H12A	109.00
C12—C13—C18	110.8 (2)	C13—C12—H12B	109.00
C14—C13—C17	100.4 (2)	H12A—C12—H12B	108.00
C13—C14—C15	110.7 (2)	C14—C15—H15	124.00
C8—C14—C13	120.8 (2)	C16—C15—H15	124.00
C8—C14—C15	128.5 (2)	C15—C16—H16A	112.00
C14—C15—C16	111.9 (3)	C15—C16—H16B	111.00
C15—C16—C17	101.6 (3)	C17—C16—H16A	112.00
C13—C17—C20	116.0 (3)	C17—C16—H16B	111.00
C16—C17—C20	118.3 (2)	H16A—C16—H16B	109.00
C13—C17—C16	104.1 (2)	C13—C17—H17	106.00
O3—C19—C10	124.6 (3)	C16—C17—H17	106.00
C17—C20—C22	124.2 (3)	C20—C17—H17	106.00
C17—C20—C21	120.1 (3)	C13—C18—H18A	109.00
C21—C20—C22	115.7 (3)	C13—C18—H18B	110.00
O5—C21—C20	123.8 (3)	C13—C18—H18C	109.00
C20—C22—C23	121.8 (3)	H18A—C18—H18B	109.00
C22—C23—C24	123.0 (3)	H18A—C18—H18C	109.00
O5—C24—C23	114.9 (3)	H18B—C18—H18C	110.00
O4—C24—O5	118.9 (4)	O3—C19—H19	118.00
O4—C24—C23	126.2 (4)	C10—C19—H19	118.00
C2—C1—H1A	109.00	O5—C21—H21	118.00
C2—C1—H1B	109.00	C20—C21—H21	118.00
C10—C1—H1A	109.00	C20—C22—H22	119.00
C10—C1—H1B	109.00	C23—C22—H22	119.00
H1A—C1—H1B	108.00	C22—C23—H23	118.00
C1—C2—H2A	109.00	C24—C23—H23	119.00
C1—C2—H2B	109.00		
			
C24—O5—C21—C20	−1.4 (8)	C11—C9—C10—C1	58.1 (3)
C21—O5—C24—O4	179.1 (5)	C11—C9—C10—C5	179.4 (2)
C21—O5—C24—C23	−0.3 (7)	C11—C9—C10—C19	−61.1 (3)
C10—C1—C2—C3	57.9 (3)	C8—C9—C11—C12	61.7 (3)
C2—C1—C10—C5	−56.2 (3)	C10—C9—C11—C12	−169.8 (2)
C2—C1—C10—C9	65.5 (3)	C1—C10—C19—O3	−14.4 (4)
C2—C1—C10—C19	−174.6 (2)	C5—C10—C19—O3	−132.5 (3)
C1—C2—C3—O1	67.1 (3)	C9—C10—C19—O3	107.6 (3)
C1—C2—C3—C4	−54.0 (3)	C9—C11—C12—C13	−58.5 (3)
O1—C3—C4—C5	−66.2 (3)	C11—C12—C13—C14	46.1 (3)
C2—C3—C4—C5	52.8 (3)	C11—C12—C13—C17	157.0 (2)
C3—C4—C5—O2	65.4 (3)	C11—C12—C13—C18	−76.8 (3)
C3—C4—C5—C6	−177.9 (3)	C12—C13—C14—C8	−42.0 (4)
C3—C4—C5—C10	−52.9 (3)	C12—C13—C14—C15	138.7 (3)
O2—C5—C6—C7	−171.8 (3)	C17—C13—C14—C8	−160.3 (2)
C4—C5—C6—C7	70.3 (3)	C17—C13—C14—C15	20.4 (3)
C10—C5—C6—C7	−54.4 (4)	C18—C13—C14—C8	81.0 (3)
O2—C5—C10—C1	−66.4 (3)	C18—C13—C14—C15	−98.3 (3)
O2—C5—C10—C9	170.4 (2)	C12—C13—C17—C16	−148.9 (2)
O2—C5—C10—C19	51.1 (3)	C12—C13—C17—C20	79.3 (3)
C4—C5—C10—C1	52.5 (3)	C14—C13—C17—C16	−31.8 (3)
C4—C5—C10—C9	−70.7 (3)	C14—C13—C17—C20	−163.6 (2)
C4—C5—C10—C19	169.9 (2)	C18—C13—C17—C16	85.7 (3)
C6—C5—C10—C1	176.8 (3)	C18—C13—C17—C20	−46.1 (3)
C6—C5—C10—C9	53.6 (3)	C8—C14—C15—C16	−179.7 (3)
C6—C5—C10—C19	−65.8 (3)	C13—C14—C15—C16	−0.4 (4)
C5—C6—C7—C8	53.6 (4)	C14—C15—C16—C17	−20.3 (4)
C6—C7—C8—C9	−52.0 (4)	C15—C16—C17—C13	31.8 (3)
C6—C7—C8—C14	−175.2 (3)	C15—C16—C17—C20	162.3 (3)
C7—C8—C9—C10	52.9 (3)	C13—C17—C20—C21	−85.2 (4)
C7—C8—C9—C11	−178.3 (3)	C13—C17—C20—C22	93.2 (4)
C14—C8—C9—C10	178.1 (2)	C16—C17—C20—C21	149.9 (4)
C14—C8—C9—C11	−53.1 (3)	C16—C17—C20—C22	−31.7 (5)
C7—C8—C14—C13	169.8 (2)	C17—C20—C21—O5	−179.0 (4)
C7—C8—C14—C15	−10.9 (4)	C22—C20—C21—O5	2.4 (7)
C9—C8—C14—C13	45.4 (3)	C17—C20—C22—C23	179.8 (4)
C9—C8—C14—C15	−135.3 (3)	C21—C20—C22—C23	−1.8 (6)
C8—C9—C10—C1	−174.6 (2)	C20—C22—C23—C24	0.1 (7)
C8—C9—C10—C5	−53.4 (3)	C22—C23—C24—O4	−178.4 (5)
C8—C9—C10—C19	66.2 (3)	C22—C23—C24—O5	0.9 (7)

### Hydrogen-bond geometry (Å, º)

**Table:**

*D*—H···*A*	*D*—H	H···*A*	*D*···*A*	*D*—H···*A*
O2—H2···O1	0.82	2.05	2.773 (3)	147
O1—H1···O2^i^	0.82	2.01	2.786 (3)	158
C9—H9···O3^ii^	0.98	2.58	3.545 (4)	169
C15—H15···O4^iii^	0.93	2.58	3.447 (4)	155
C22—H22···O4^iv^	0.93	2.60	3.233 (6)	126

Symmetry codes: (i) −*x*+1, *y*−1/2, −*z*+2; (ii) *x*, *y*−1, *z*; (iii) *x*+1, *y*, *z*; (iv) −*x*−1, *y*+1/2, −*z*+1.
